# Supplementary material for: Modelling Skylarks (Alauda arvensis) to Predict Impacts of Changes in Land Management and Policy: Development and Testing of an Agent-Based Model
Source: PLoS One. 2013 Jun 6;8(6):e65803. doi: 10.1371/journal.pone.0065803 (PMC3675089; doi:10.1371/journal.pone.0065803)
Supplement: Supporting Information S4 — The skylark ODdox as a zipped archive. (ZIP) [file pone.0065803.s004.zip › Skylark_ODdox/classprobe__data.html]

ALMaSS Skylark ODdox: probe\_data Class Reference


ALMaSS Skylark ODdox
 2.0


- Main Page
- Related Pages
- Classes
- Files

- Class List
- Class Index
- Class Hierarchy
- Class Members

Public Member Functions |
Public Attributes |
Protected Attributes

probe\_data Class Reference

Data structure to hold & output probe data probe data is designed to be used to return the number of objects in a given area or areas in specific element or vegetation types or farms.
More...

`#include <populationmanager.h>`

List of all members.

|  |  |
| --- | --- |
| Public Member Functions | |
| void | CloseFile () |
| void | FileAppendOutput (int No, int time) |
| void | FileOutput (int No, int time, int ProbeNo) |
| ofstream \* | OpenFile (char \*Nme) |
| bool | OpenForAppendToFile () |
|  | probe\_data () |
| void | SetFile (ofstream \*F) |
|  | ~probe\_data () |

|  |  |
| --- | --- |
| Public Attributes | |
| bool | m\_FileRecord |
| unsigned | m\_NoAreas |
| unsigned | m\_NoEleTypes |
| unsigned | m\_NoFarms |
| unsigned | m\_NoVegTypes |
| rectangle | m\_Rect [10] |
| TTypesOfLandscapeElement | m\_RefEle [25] |
| unsigned | m\_RefFarms [25] |
| TTypesOfVegetation | m\_RefVeg [25] |
| unsigned | m\_ReportInterval |
| bool | m\_TargetTypes [10] |

|  |  |
| --- | --- |
| Protected Attributes | |
| ofstream \* | m\_MyFile |
| char | m\_MyFileName [255] |
| int | m\_Time |

---

## Detailed Description

Data structure to hold & output probe data probe data is designed to be used to return the number of objects in a given area or areas in specific element or vegetation types or farms.

---

## Constructor & Destructor Documentation

|  |  |  |  |  |
| --- | --- | --- | --- | --- |
| probe\_data::probe\_data | ( |  | ) |  |

Constructor for probe\_data

{

m\_Time = 0;

m\_FileRecord = false;

m\_MyFile = NULL;

}

|  |  |  |  |  |
| --- | --- | --- | --- | --- |
| probe\_data::~probe\_data | ( |  | ) |  |

Destructor for probe\_data

{

}

---

## Member Function Documentation

|  |  |  |  |  |
| --- | --- | --- | --- | --- |
| void probe\_data::CloseFile | ( |  | ) |  |

Referenced by CloseDownSim().

{

if ( m\_MyFile != NULL ) {

if (m\_MyFile->is\_open()) m\_MyFile->close();

delete m\_MyFile;

}

};

|  |  |  |  |
| --- | --- | --- | --- |
| void probe\_data::FileAppendOutput | ( | int | *No*, |
|  |  | int | *time* |
|  | ) |  |  |

References g\_msg, and MapErrorMsg::Warn().

{

m\_MyFile->open(m\_MyFileName,ios::app);

if ( !m\_MyFile->is\_open() ) {

g\_msg->Warn( (MapErrorState)0,"Cannot open file for append: ", m\_MyFileName );

exit( 0 );

}

if ( m\_FileRecord ) {

(\*m\_MyFile)<< time << '\t' << No << endl;

}

m\_MyFile->close();

}

|  |  |  |  |
| --- | --- | --- | --- |
| void probe\_data::FileOutput | ( | int | *No*, |
|  |  | int | *time*, |
|  |  | int | *ProbeNo* |
|  | ) |  |  |

Basic output function of the default probe data file.   
This just counts numbers in specified areas

Referenced by Skylark\_Population\_Manager::ProbeReportPOM().

{

if ( m\_FileRecord ) {

if ( ProbeNo == 0 ) {

// First probe so write the time and a new line

(\*m\_MyFile) << endl;

(\*m\_MyFile) << time << '\t' << No;

} else

(\*m\_MyFile) << '\t' << No ;

}

(\*m\_MyFile).flush();

}

|  |  |  |  |  |  |
| --- | --- | --- | --- | --- | --- |
| ofstream \* probe\_data::OpenFile | ( | char \* | *Nme* | ) |  |

Opens the default probe data output file

References g\_msg, and MapErrorMsg::Warn().

Referenced by GetProbeInput\_ini().

{

m\_MyFile = new ofstream( Nme );

if ( !m\_MyFile->is\_open() ) {

g\_msg->Warn( (MapErrorState)0,"probe\_data::OpenFile - Cannot open file: ", Nme );

exit( 0 );

}

strcpy( m\_MyFileName, Nme );

return m\_MyFile;

}

|  |  |  |  |  |
| --- | --- | --- | --- | --- |
| bool probe\_data::OpenForAppendToFile | ( |  | ) |  |

inline

References g\_msg, m\_MyFile, m\_MyFileName, MapErrorMsg::Warn(), and WARN\_FILE.

{

m\_MyFile = new ofstream(m\_MyFileName, ios::app);

if (!(\*m\_MyFile).is\_open())

{

g\_msg->Warn( WARN\_FILE, "PopulationManager::AppendToFile() Unable to open file for append: ", m\_MyFileName );

exit(1);

}

return true;

}

|  |  |  |  |  |  |
| --- | --- | --- | --- | --- | --- |
| void probe\_data::SetFile | ( | ofstream \* | *F* | ) |  |

Sets the filename for the default probe data output

Referenced by GetProbeInput\_ini().

{

m\_MyFile = F;

}

---

## Member Data Documentation

bool probe\_data::m\_FileRecord

ofstream\* probe\_data::m\_MyFile

protected

Referenced by OpenForAppendToFile().

char probe\_data::m\_MyFileName[255]

protected

Referenced by OpenForAppendToFile().

unsigned probe\_data::m\_NoAreas

Referenced by TSkylarkList< T >::Probe(), and Population\_Manager::Probe().

unsigned probe\_data::m\_NoEleTypes

Referenced by TSkylarkList< T >::Probe(), and Population\_Manager::Probe().

unsigned probe\_data::m\_NoFarms

Referenced by TSkylarkList< T >::Probe(), Population\_Manager::Probe(), and Skylark\_Population\_Manager::ProbePOM().

unsigned probe\_data::m\_NoVegTypes

Referenced by TSkylarkList< T >::Probe(), and Population\_Manager::Probe().

rectangle probe\_data::m\_Rect[10]

Referenced by TSkylarkList< T >::Probe(), and Population\_Manager::Probe().

TTypesOfLandscapeElement probe\_data::m\_RefEle[25]

Referenced by TSkylarkList< T >::Probe(), and Population\_Manager::Probe().

unsigned probe\_data::m\_RefFarms[25]

Referenced by TSkylarkList< T >::Probe(), Population\_Manager::Probe(), and Skylark\_Population\_Manager::ProbePOM().

TTypesOfVegetation probe\_data::m\_RefVeg[25]

Referenced by TSkylarkList< T >::Probe(), and Population\_Manager::Probe().

unsigned probe\_data::m\_ReportInterval

bool probe\_data::m\_TargetTypes[10]

int probe\_data::m\_Time

protected

---

The documentation for this class was generated from the following files:

- populationmanager.h
- PopulationManager.cpp


- probe\_data
- Generated on Thu Jan 10 2013 13:15:37 for ALMaSS Skylark ODdox by
   1.8.1.1
